# Men who have sex with men sensitivity training reduces homoprejudice and increases knowledge among Kenyan healthcare providers in coastal Kenya

**DOI:** 10.7448/IAS.16.4.18748

**Published:** 2013-12-02

**Authors:** Elise M van der Elst, Adrian D Smith, Evanson Gichuru, Elizabeth Wahome, Helgar Musyoki, Nicolas Muraguri, Greg Fegan, Zoe Duby, Linda-Gail Bekker, Bonnie Bender, Susan M Graham, Don Operario, Eduard J Sanders

**Affiliations:** 1 Kenya Medical Research Institute-Wellcome Trust Research Programme, Kilifi, Kenya; 2 Department of Public Health, University of Oxford, Oxford, UK; 3 National AIDS and STI Control Programme, Nairobi, Kenya; 4 Desmond Tutu HIV Foundation, Cape Town, South Africa; 5 International AIDS Vaccine Initiative, Nairobi, Kenya; 6 Department of Global Health, University of Washington, Seattle, WA, USA; 7 Department of Community Health, Brown University, Providence, RI, USA

**Keywords:** sensitivity training, MSM behaviour, Homophobia Scale, homoprejudice, healthcare workers, Kenya

## Abstract

**Introduction:**

Healthcare workers (HCWs) in Africa typically receive little or no training in the healthcare needs of men who have sex with men (MSM), limiting the effectiveness and reach of population-based HIV control measures among this group. We assessed the effect of a web-based, self-directed sensitivity training on MSM for HCWs (www.marps-africa.org), combined with facilitated group discussions on knowledge and homophobic attitudes among HCWs in four districts of coastal Kenya.

**Methods:**

We trained four district “AIDS coordinators” to provide a two-day training to local HCWs working at antiretroviral therapy-providing facilities in coastal Kenya. Self-directed learning supported by group discussions focused on MSM sexual risk practices, HIV prevention and healthcare needs. Knowledge was assessed prior to training, immediately after training and three months after training. The Homophobia Scale assessed homophobic attitudes and was measured before and three months after training.

**Results:**

Seventy-four HCWs (68% female; 74% clinical officers or nurses; 84% working in government facilities) from 49 health facilities were trained, of whom 71 (96%) completed all measures. At baseline, few HCWs reported any prior training on MSM anal sexual practices, and most HCWs had limited knowledge of MSM sexual health needs. Homophobic attitudes were most pronounced among HCWs who were male, under 30 years of age, and working in clinical roles or government facilities. Three months after training, more HCWs had adequate knowledge compared to baseline (49% vs. 13%, McNemar's test *p*<0.001); this was most pronounced in those with clinical or administrative roles and in those from governmental health providers. Compared to baseline, homophobic attitudes had decreased significantly three months after training, particularly among HCWs with high homophobia scores at baseline, and there was some evidence of correlation between improvements in knowledge and reduction in homophobic sentiment.

**Conclusions:**

Scaling up MSM sensitivity training for African HCWs is likely to be a timely, effective and practical means to improve relevant sexual health knowledge and reduce personal homophobic sentiment among HCWs involved in HIV prevention, testing and care in sub-Saharan Africa.

## Introduction

Sub-Saharan Africa has a very high burden of HIV-1 infection, of which a substantial proportion occurs among populations reporting high-risk sexual behaviour such as transactional sex and anal intercourse [1]. Such populations suffer from stigma and rejection, and they have been neglected by many HIV prevention and care programmes [2]. As a result, most African healthcare workers (HCWs) have not been informed about the risk of HIV transmission with regard to heterosexual or homosexual anal sex. In addition, African HCWs may lack understanding of the many challenges that men who have sex with men (MSM) and other key populations face in healthcare facilities [3].

Societal discrimination on the grounds of sexual orientation has been reported frequently among African MSM, taking the form of sexual, physical and verbal assault [4–7], and a number of studies have demonstrated an association between reported experience of discrimination and HIV risk or risk behaviour [8]. Similarly, high levels of internalized homophobia among MSM have been reported in Nigeria [8], South Africa [9,10] and Uganda [11], known to be associated with individual HIV risk-taking behaviour [12]. Overt stigmatization specifically from HCWs in the context of HIV testing and care, such as denial of service [4,3,13] and harassment in clinic spaces [14], has been reported as a key element of perceived discrimination, presenting a deterrent to service access [15] or accurate disclosure of behavioural risk [14]. In the absence of resources targeted to groups at high risk of HIV infection, the marginalization of MSM from public HIV prevention and treatment resources can only hamper the effectiveness of national HIV control efforts [6].

Health worker training, social mobilization and community engagement were prioritized as structural interventions in a recent consultation on priority areas for MSM HIV prevention research involving 69 participants from 17 African countries [16]. HCWs have also been *called to action* to reduce stigma and discrimination, provide integrated services for mental health concerns and substance use, screen MSM routinely for HIV and sexually transmitted infections (STIs) and ensure training for all personnel in clinical settings [3]. As yet, African HCWs lack any evidence-based, culturally adapted training model that is sensitive to MSM needs. This problem likely stems from cultural taboos about anal sex practices, even in opposite-sex couples [17], and strong political, religious and public prejudice against same-sex practices [18].

Since 2005, biomedical research has been ongoing with both HIV-1 negative MSM and MSM living with HIV in coastal Kenya [1,19]. To date, the only incidence data for African MSM derive from our cohort and a related cohort in Nairobi [19,20]. Overall HIV-1 incidence among young MSM in coastal Kenya was as high as 8.6 (95% confidence interval [CI]: 6.7–11.0) per 100 person-years of observation [19]. The majority of these MSM reported sex work, and large numbers of such men have been identified in coastal Kenya [21]. Similarly, our cohort study of MSM living with HIV showed that 40% had less than 95% antiretroviral therapy (ART) adherence, compared to 29% of heterosexual men and 12% of women who were followed in the same research setting [1]. These findings prompted us to brief health authorities and develop materials to help improve care for MSM in Kenya and elsewhere in Africa.

Internet-based learning (e-learning) as a cognitive tool has increasingly been used in health professions in resource-constrained low- and middle-income countries [22]. E-learning technologies offer learners control over content, learning sequence, pace of learning, time and often media, allowing learners to tailor their experiences to meet their personal learning objectives [23]. The internet-based HCW MSM sensitivity training described here represents our attempt to deploy meaningful, clinically relevant material to meet this specific learning need within Kenyan HIV services through adaptation of an existing training curriculum to a web environment.

“MSM: An introductory guide for health workers in Africa” is a paper-based HCW sensitization training first developed in 2010. The content of training was validated and revised through a programme of extensive classroom use in South Africa, and following expert review [24]. The paper-based training guide was electronically converted to a self-directed electronic format and published online in July 2011, a version of which was adapted for use in this study.

The objectives of this study were (1) to assess the feasibility of facilitated self-directed learning of MSM health issues in coastal Kenya and (2) to evaluate the effect of the training intervention upon HCW knowledge and attitudes.

## Methods

### Study site and participants

Seventy-four HCWs involved in HIV prevention, treatment and care services in coastal Kenya were recruited to participate in the study. We mapped 54 ART-providing governmental and nongovernmental health providers in four districts in coastal Kenya (Kilindini, Mombasa, Kilifi and Malindi). An average of two staff representatives from each health-providing facility were invited to the training intervention, including clinicians and counsellors as well as clinic administrators.

Four “district AIDS/STD coordinators” (DASCOs) working within the study districts were trained to lead the MSM sensitization training during a 2-day “training-of-trainers” course similar to the training proper. An additional day was used to prepare focus-group topic guides and organizational matters. The study procedures were approved by the ethical review board at the Kenya Medical Research Institute, and all participants provided written informed consent for impact evaluation. HCWs received Ksh2000 (approximately US$24.00) to cover travel expenses and lodging.

### The training intervention

The training consisted of two consecutive days and included eight modules which were taken in four sessions (i.e., two computer modules per session). Each session was followed by a group discussion. Each group size comprised 18–19 participants. DASCOs were supported by four members of the research team (i.e., a community liaison officer, a research counsellor, an MSM staff-fieldworker and a social scientist) and two members of a local LGBTI (lesbian, gay, bisexual, transgender and intersex) organization. HCWs were introduced to the sensitivity training on MSM health issues and learned that the training consisted of computer-assisted learning (http://www.marps-africa.org) and group discussions. The curriculum consisted of the following modules of study: (1) *MSM and HIV in sub-Saharan Africa*; (2) *Stigma*; (3) *Identity, coming out and disclosure*; (4) *Anal sex and common sexual practices*; (5) *HIV and sexually transmitted infections*; (6) *Mental health, anxiety, depression and substance abuse*; (7) *Condom and lubricant use*; and (8) *Risk reduction counselling*. Modules were designed to be self-completed in 1–2 hours each, including multiple-choice questions (median 12, range 9–16) at the end of each module. A score of 71% correct was required to advance to the next module, and upon successful completion of all eight modules, participants were sent a link to download their course certificate. A post-course evaluation asked for opinions and suggestions for course improvements, using both closed and open-ended questions.

Discussion topics included the identification of subcategories of MSM and their characteristics, sexual practices of MSM and risks for HIV and STI transmission, factors that make MSM vulnerable to STIs and HIV, risk assessment in counselling MSM, best practice for sexual history taking and sexual health examination with MSM, relevant information on safer sex for MSM, personal values and attitudes towards MSM, and addressed stigma and strategies to improve communication with clients who are MSM. At the end of the training, HCWs discussed a work plan on how to strengthen clinical care and uptake of HIV and STD testing for MSM in their day-to-day practice. Study participants with a clinical role were also requested to keep a journal for three months to document and reflect upon their work practices and personal attitudes towards MSM.

### Data collection

Course participants completed an online registration, including socio-demographic characteristics (age, gender and level of education and training), details of working practice (role within, type and location of healthcare organization) and specific experience working with HIV prevention, treatment and care with the most at-risk populations (MARPs) in Africa. To assess baseline levels of knowledge, participants conducted a pre-course 24-item multiple-choice assessment covering key learning outcomes across the course material, and they completed a 25-item Homophobia Scale (HS; adapted from Wright et al. [25]). The same two measures were repeated three months after course completion to assess sustained changes in knowledge of and attitudes towards MSM. Immediate post-course knowledge was assessed using the same pre-knowledge questionnaire upon completion of the eight modules. The results of pre-training and post-training assessments were not communicated to participating HCWs.

### Measurement scales

Knowledge scores of course material were divided into the following categories: Poor (<17 questions correctly answered),Good (17–22 questions correctly answered) and Excellent (>22 questions correctly answered). When 17 or more questions were correctly answered, the immediate post-training knowledge was considered adequate.

The HS, which was developed and standardized among college students in the United States by Wright et al. [25], was adapted for use in Kenya. The HS aims to measure thoughts, feelings and behaviours towards homosexuality and MSM, and it consists of 25 statements to which respondents indicate their level of agreement on a 5-point Likert scale (Table 1). Questions were reviewed and adapted by three Kenyan research staff and HCWs with professional and personal experience working with local MSM. The adapted HS is shown in Table 1 and reflects changes in terminology (e.g., “gay” was replaced with “MSM” and “faggot” with “shoga” in question 9) to reflect local terminology in current use. *I have damaged property of gay persons, such as “keying” their cars*, was replaced with *Homosexuality should be treated as an illness/Homosexuality can be cured* (question 17); *I would feel comfortable with having a gay roommate* was replaced with *Homosexuality is un-African/is something brought by foreigners* (question 18); and *I have rocky relationships with people that I suspect are gay* was replaced with *Gay men have the same rights to public/tax-funded services as straight men* (question 25). Responses to items 1, 2, 4, 5, 6, 9, 12, 13, 14, 15, 17, 18, 19, 21, 23 and 24 were reverse coded (item scores 1=5, 2=4, 3=3 etc.). The total HS score (HSS) was the sum of all item scores, with 25 subtracted from the total. The range is between 0 and 100, with an HSS of 0 being the least homophobic and 100 being the most homophobic.

**Table 1:** Homophobia Scale and MSM sensitivity training for healthcare workers (HCWs), coastal Kenya, 2011–2012

***This questionnaire is designed to measure your thoughts, feelings and behaviours with regards to homosexuality. It is not a test, so there are no right or wrong answers. Answer each item by circling the number after each question**.*						
**1=Strongly agree**						
**2=Agree**						
**3=Neither agree nor disagree**						
**4=Disagree**						
**5=Strongly disagree**						

1	MSM make me nervous	1	2	3	4	5
2	MSM deserve what they get	1	2	3	4	5
3	Homosexuality is acceptable to me	1	2	3	4	5
4	If I discovered a friend was an MSM, I would end the friendship	1	2	3	4	5
5	I think homosexual people should not work with children	1	2	3	4	5
6	I make derogatory remarks about MSM people	1	2	3	4	5
7	I enjoy the company of MSM	1	2	3	4	5
8	Marriage between homosexual individuals is acceptable	1	2	3	4	5
9	I make derogatory remarks like “shoga” or “queer” to people who I suspect are MSM	1	2	3	4	5
10	It does not matter to me whether my friends are MSM or straight	1	2	3	4	5
11	It would upset me if I learned that a close friend was homosexual	1	2	3	4	5
12	Homosexuality is immoral	1	2	3	4	5
13	I tease and make jokes about MSM	1	2	3	4	5
14	I feel that you cannot trust a person who is homosexual	1	2	3	4	5
15	I fear homosexual persons will make sexual advances towards me	1	2	3	4	5
16	Organizations which promote gay rights are not necessary	1	2	3	4	5
17	Homosexuality should be treated as an illness/Homosexuality can be cured	1	2	3	4	5
18	Homosexuality is un-African/is something brought by foreigners	1	2	3	4	5
19	I would hit a homosexual for coming on to me	1	2	3	4	5
20	Homosexual behaviour should not be against the law	1	2	3	4	5
21	I avoid MSM individuals	1	2	3	4	5
22	It bothers me to see two homosexual people together in public	1	2	3	4	5
23	When I see an MSM, I think, “What a waste.”	1	2	3	4	5
24	When I meet someone, I try to find out if he or she is MSM	1	2	3	4	5
25	Gay men have the same rights to public/tax-funded services as straight men	1	2	3	4	5

### Data analysis

Analysis was conducted using Stata 11.0 (StataCorp LP, College Station, TX, USA). Binary and categorical characteristics of study participants, established at baseline, were compared using chi-square tests. Although both knowledge and HS scores before and after training approximated to Gaussian distributions, differences between paired measures were non-normal, and thus unadjusted nonparametric methods were used for analysis. Median differences between pre- and post-training knowledge and homophobia score are reported with an interquartile range (IQR). A Wilcoxon signed rank test for matched pairs was applied to test the statistical significance of differences between pre- and post-training scores. Mann–Whitney and exact McNemar's tests were used to test differences in scores and binary measures, respectively, by HCW characteristics. Spearman's rank was used to assess correlation between pre- and post-training scores, and knowledge and HS scores at both points. Multivariate linear regression models of pre- and post-training score outcomes were explored, but they yielded no additional insight beyond bivariate analysis.

## Results

Seventy-four HCWs were recruited to participate in the training programme, and their characteristics are shown in Table 2. The majority were female, and the mean age of participants was 32 years (range: 23 to 53). Sixty-two participants (84%) worked at a government health facility (hospital or clinic), seven (9%) worked at a local nongovernmental organization (NGO) and three (4%) represented faith-based organizations. Most (74%) were in a clinical role (nurse or clinical officer). Irrespective of job role, 8% had received any previous training on how to counsel MSM clients, and a similarly low proportion (7%) had ever received training on how to counsel on anal sex practices. HCWs who had received training on anal sex practices were more likely to have ever asked their male patients if they had sex with men than HCWs who did not report previous training (86% (6/7) versus 31% (21/67), χ^2^ *p*<0.01).

**Table 2:** Characteristics of 74 healthcare workers (HCWs)

Registration characteristics	HCWs selected for two-day online training *N*=74 *N* (%)
Gender	
Men	24 (32)
Female	50 (68)
Age (years)	
18–29	3 (4)
30–39	44 (59)
40 +	27 (36)
Organization type	
Government	62 (84)
NGO	7 (9)
Other	5 (7)
Education level	
Up to secondary	6 (8)
Tertiary	68 (92)
Job type	
Clinical	52 (70)
Counselling	12 (16)
Administrative	7 (9)
Other	3 (4)
**Prior MSM training experience**	
Counselling clients who are MSM	
No	68 (92)
Yes	6 (8)
Counselling on anal sex practices	
No	67 (91)
Yes	7 (9)
Ever asked male clients about sexual acts with other men	
No	47 (64)
Yes	27 (36)

### Training logistics

Most HCWs (73% (54/74)) required 1 hour or less to complete each module, whilst 11% (8/74) required 2 hours or more per module. While 68% (50/74) considered the course duration to be “just right,” 23% (17/74) said that it was too short, and 9% (7/74) that it was too long. Overall, by the end of training, 60/74 (81%) HCWs reported feeling empowered to discuss MSM behaviour and anal sex in their professional work.

All participants said they would recommend the course to others. Open-ended suggestions for course improvements are presented in Table 3. Study participants recommended that the training should be taken by all health stakeholders dealing with MSM issues and be included in medical training. There was an interest in similar training related to other key populations (e.g., women who have sex with women and sex workers).

**Table 3:** End-of-course suggestions for course improvements

**Study participants (SP) (** ***N*** **=74)**
Selected from 45 (61%) responses
**Theme: Expand training to all healthcare workers (HCWs) and other institutions**
“This online training should be streamed or provided to all institutions and politicians to sensitize them on the need to recognize MSM.”
“Should be introduced in medical training departments.”
“It should be done by all health stake holders dealing with MSM issues.”
**Theme: Advertisement and promotion website**
“Reach more people through media eg Radio and schools.”
“Make it available at facility by providing computer.”
**Theme: Connectivity, internet, computers and mobile phones**
“Improve internet speed.”
“No suggestion, it is that I am still learning how to use a computer.”
**Theme: Duration of course**
“It should also include field work.”
“The course is very interesting, it should be for at least five days, so we get time to discuss more.”
(Nineteen additional participants asked for a longer course.)
**Theme: Improve specific modules and further learning**
“Add women who have sex with women/prostitution.”
“Add more pictures on the STI module. Make the questions more relevant for some are contradictory. Have authoritative literature references.”
“The picture of the actual penetration on page 68 [cartoon of man penetrating a man while using a condom] is somehow too much to view. Better picture can be used to demonstrate the same.”
“We need more testimony clips to narrate how many MSM got to fight stigma and be where they are now.”
“More research should be conducted on whether homosexuality could be reversed.”
“Perhaps translation in Kiswahili.”

### Effect of training on MSM sexual health knowledge among healthcare workers

Table 4 shows knowledge of MSM sexual health issues among participants before the training course, and upon reassessment three months after the course. Prior to the training course, only 10/74 (14%) had an “adequate” level of knowledge of MSM issues (threshold score: 17/24), reflecting a median score of 54% (IQR 49–63%). Levels of knowledge were similar by socio-demographic and workplace characteristics of HCWs, although it was somewhat lower for HCWs in administrative roles compared to other roles (median 42 vs. 54, Mann–Whitney *p*=0.293).

**Table 4:** Change in MSM sexual health knowledge from baseline to three months post-training

					Difference between pre-training and post-training multiple-choice questions %†		
					
		Healthcare workers (HCWs) *N*	Pre-training (baseline) Median (%)	Post-training (three month) Median (%)	Median difference (%)	Interquartile range	*P*-value (Wilcoxon)
All		74	54	67	+12	4 to 21	<0.001
Gender	Male	24	56	71	+16	5 to 21	<0.001
	Female	50	54	65	+11	1 to 21	<0.001
	Under 30	23	54	71	+16	0 to 21	0.001
Age group	30 to 39	34	58	63	+9	0 to 21	0.002
	40 or over	17	54	71	+12	4 to 21	0.002
	Clinical	52	54	71	+13	8 to 21	<0.001
	Counselling	12	58	56	0	−9 to 13	0.813
Job type	Admin	7	42	75	+13	4 to 37	0.022
	Other	3	54	73	+19	9 to 29	0.190
	Government	62	54	71	+13	5 to 21	<0.001
Facility	NGO	7	58	67	+12	−8 to 17	0.267
	Other	5	54	58	+4	−5 to 25	0.414

† Limited to 71 HCWs with paired observations.

At the end of training, 70/74 (95%) HCWs had adequate course knowledge (exact McNemar's χ^2^ *p*<0.001 vs. pre-training). At three months after the course, 35 (49%) of the 71 HCWs reassessed had retained “adequate” knowledge compared to 9/71 (13%) at pre-training (exact McNemar's χ^2^ *p*<0.001). This represented a significant increase in the median assessment score of 12% (IQR 4–21%) between baseline and three-month knowledge assessments (Wilcoxon signed test for matched pairs *p*<0.001). Significant sustained improvements in knowledge were apparent for all HCW age groups and genders, those with clinical or administrative roles and those from governmental health providers.

Pre-training and three-month post-training scores were negatively correlated (Spearman's rho −0.51, *p<* 0.001), indicating that improvements in knowledge tended to be highest among HCWs with lower pre-training knowledge. There were no significant differences in the degree of knowledge gain by the gender or age group of HCWs; however, participants in counselling roles achieved significantly lower gains in sustained knowledge than other HCWs (median difference: 0% vs. +13%, Mann–Whitney *p*=0.0163).

### Effect of training on personal attitudes toward MSM among healthcare workers

Table 5 shows HS scores among HCWs prior to training and at reassessment three months later. Overall, the median HS score prior to training was 68/100, representing extensive agreement with homophobic statements and disagreement with statements indicating tolerance of MSM (see Table 5). Male HCWs had slightly higher HS scores at baseline than female HCWs, while HS scores declined with increasing age group but differences were not statistically significant. HCWs in clinical roles (medical and nursing) had higher HS scores than other staff (median 71 vs. 66 respectively, Mann–Whitney *p*=0.116), and HCWs working in government facilities had significantly higher HS scores than HCWs in NGOs (Table 5, Mann–Whitney *p*=0.037).

**Table 5:** Change in Homophobia Score (HS) from baseline to three months post-training

					Difference between pre-training and post-training HS†		
					
		Healthcare workers (HCWs) *N*	Pre-training (baseline) Median HS (0–100)	Post-training (three month) Median HS score (0–100)	Median difference (score)	Interquartile range	*P*-value (Wilcoxon)
All		74	68	60	−8	−2 to −15	<0.001
Gender	Male	24	70	60	−9	−4 to −21	0.001
	Female	50	68	60	−7	−1 to −13	<0.001
	Under 30	23	71	62	−9	−3 to −14	<0.001
Age group	30 to 39	34	68	59	−6	−1 to −13	<0.001
	40 or over	17	67	61	−10	−4 to −24	0.001
	Clinical	52	71	61	−10	−3 to −20	<0.001
Job type	Counselling	12	64	60	−5	+1 to −9	0.031
	Administrative	7	68	55	−11	−6 to −19	0.022
	Other	3	66	61	−2	0 to −4	0.317
	Government	62	71	60	−9	−2 to −15	<0.001
Facility	Nongovernmental organization	7	62	56	0	+1 to −13	0.444
	Other	5	78	60	−9	−5 to −21	0.043

† Limited to 71 HCWs with paired observations.

**Figure 1:**
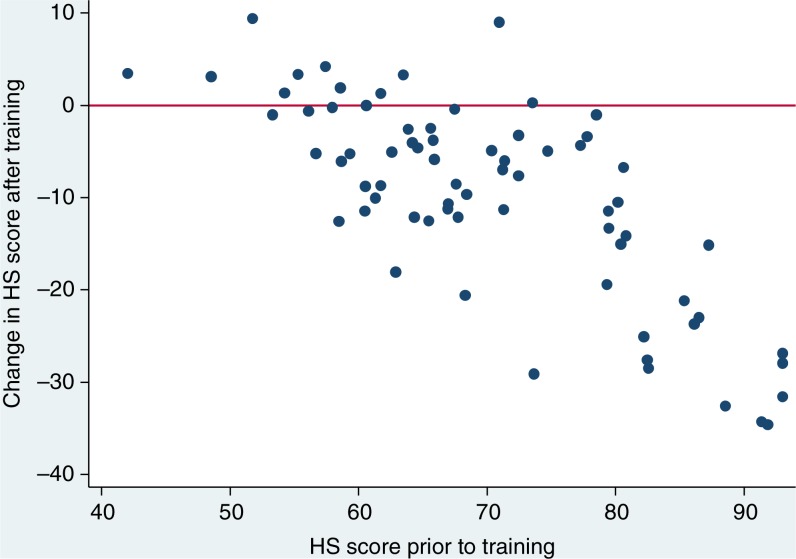
Baseline HS score and difference three months after training (71 participants).

The majority of HCWs reported lower HS scores three months post-training (80.3%, 57/71) compared to their baseline HS score; in four (5.6%), HS scores were unchanged; and in 14.1% (10/71), HS scores were higher after training than before. Overall, the median decrease in individual HS score after training was 8 points (IQR 2–15), which was statistically significant. These findings did not change in a sensitivity analysis omitting the three HS questions that were culturally adapted (data not shown). Individual pre-training and post-training HS scores were negatively correlated (Spearman's rho=− 0.71, *p*<0.001), reflecting the tendency for HCWs with high pre-training HS scores to exhibit greater decreases in this measure as a result of training (Figure 1).

Male HCWs and those working in clinical roles and in governmental institutions recorded the most pronounced reductions in HS score subsequent to training, although differences in median reduction comparing HCWs’ gender, age group, staff role and institution were not statistically significant. More modest declines in HS score were apparent for counsellors (median reduction after training: 4 points) and staff of NGOs (median reduction after training: 0 points); however, it is notable that these groups reported relatively low HS scores prior to training. Collectively, there was some evidence for correlation between scale of increase in individual knowledge and scale of decline in HS score, and this was of borderline statistical significance (Spearman's rho=−.21, *p*=0.087).

## Discussion

This formal evaluation of a training course aimed specifically to improve knowledge and awareness of MSM sexual health needs among healthcare staff involved in frontline HIV prevention, treatment and care to adult populations in sub-Saharan Africa. Specific and accurate knowledge relevant to the management of behavioural and clinical risks for MSM clients prior to training was poor.

Whilst this may not be surprising in the face of long-standing neglect of Kenyan MSM within HIV policy and resource allocation and a lack of attention to MSM within medical, nursing and HIV counselling training in Kenya, it draws focus to the challenge of maintaining and extending the professional competence of the existing HIV workforce to match the epidemiological realities of the Kenyan HIV epidemic – especially since the National AIDS & Sexually Transmitted Diseases Control Programme (NASCOP) requires Kenyan HCWs to document the number and category of MSM using HIV services.

Whilst targeted services may well be necessary for subpopulations of MSM, such as male sex workers, they are unlikely to replace the need for MSM-specific clinical care among general health services. MSM-specific programmes have aroused considerable social antipathy in Kenya to date [26] and may in any case not be perceived as accessible to men who covertly engage in homosexual behaviour [27]. Furthermore, surveillance of key populations, including MSM, and strategic information on service coverage to these groups are now an international requirement [28].

The combination of self-directed, modular computer-based learning supplemented by group discussions facilitated by trainers identified from within the existing workforce may offer a relatively sustainable and mobile model for episodic health professional training in this context. The learning content of this course is freely available as a web resource, yet reliable access to internet services remains elusive and expensive in most parts of the country. Even where it is available, the narrative reflections by participants who undertook this training emphasize the importance of the sanction provided by facilitated group discussions to share and explore personal and professional issues arising from the training content that may well be lost in self-directed learning [29].

The brief training programme described here resulted in significant improvements in knowledge of MSM sexual health issues pertinent to day-to-day prevention and clinical practice, and it was sustained by most trainees until at least three months after training. Increase in knowledge was accompanied by a reduction in negative attitudes toward MSM over the same period. Encouragingly, the positive effect of training upon knowledge and personal attitudes toward MSM was strongest among HCWs who had poor levels of knowledge and/or more extreme negative attitudes toward MSM prior to training. That positive changes were most marked among HCWs in clinical roles within governmental settings, which represent the backbone of Kenyan HIV services, is cause for particular optimism. Studies to date of perceived barriers to healthcare access identified by MSM in Kenya [7,30] and elsewhere in sub-Saharan Africa [31,32] have reported denial of service, lack of confidentiality, ignorance and verbal abuse from governmental HIV services as central challenges in accessing sexual and general health services. The finding of this study, albeit preliminary, suggests both that members of this workforce are willing to learn about MSM sexual health and that their knowledge and attitude toward MSM are responsive to this learning.

This study has a number of limitations. The HS, which was originally developed and validated among college students in the United States [25], required amendment to preserve its face validity in a markedly different research context. Whereas the modified scale was responsive to change with training, and these changes were robust to sensitivity analysis excluding modified scale items, the objective meaning of absolute scores and the convergent and divergent validity of this scale in this population remain to be established. Furthermore, although the assessment of training effects upon knowledge and homophobic sentiment was assessed at an endpoint long after the training itself, the longer term effect of training cannot be assumed from this study. Finally, this study lacked a control group which, ideally, would have consisted of HCWs not receiving the intervention and HCWs only participating in the self-directed learning.

We report qualitative narratives among HCWs returning to their workplace after training but finding little support for new perspectives amongst (untrained) colleagues [29]. In a recent qualitative assessment of counselling challenges regarding MSM that are experienced by Kenyan counsellors and clinicians in coastal Kenya, all felt that lack of training and supervisory support impacted their ability to serve MSM [33]. These findings may suggest that either longer term support of trained HCWs and/or more extensive facility-based training of all staff may be prerequisites to longer term changes in institutional practice. While knowledge of same-sex practices is a first step to improve services to MSM, serving MSM in day-to-day practice will further improve services. Follow-up of health workers trained in this study is planned and may provide insights in current care services provided to MSM at two years post-training. Additionally, although the training itself was conducted by facilitators from governmental services that were specially trained for the role, the study was run by a team who was unusually experienced in working with Kenyan MSM, which may threaten generalizability to other settings.

Finally, the ultimate goals of improving knowledge of MSM sexual health needs and reducing prejudicial attitudes toward MSM in healthcare settings are to enhance the accessibility of population-based, public health services to MSM themselves. Although surely a prerequisite to accessible HIV prevention, treatment and care for MSM, the extent to which changes in the attitudes and practices of healthcare providers are reflected in the perceived and practical accessibility and acceptability of services to MSM themselves is unknown. Further study will be required to establish the effect of this brief intervention on long-term attitudes and professional practices towards MSM, and what practical contribution such strategies might make to addressing unmet HIV-related needs among MSM.

## Conclusions

In summary, we developed, implemented and evaluated a brief training intervention addressing knowledge and attitudes toward MSM and their sexual health needs in Kenya. The training, which combined self-directed and facilitated group learning, increased health worker knowledge and reduced homophobic attitudes up to three months after training. Scaling up such interventions offers a straightforward response to the immediate need to support HCWs in offering accessible and informed services to address the largely sexual health needs among MSM in Kenya.
